# An Integrated Fuzzy AHP and Fuzzy TOPSIS Approach to Assess Sustainable Urban Development in an Emerging Economy

**DOI:** 10.3390/ijerph16162902

**Published:** 2019-08-13

**Authors:** Van Thac Dang, Jianming Wang, Wilson Van-Thac Dang

**Affiliations:** 1 Department of Business Administration, Business School, Shantou University, Guangdong 515063, China; 2 School of Business Administration, Zhejiang University of Finance & Economics, Hangzhou 310018, China; 3 Dong Nai Technology University, Bien Hoa, Dong Nai 810000, Vietnam

**Keywords:** sustainable development, sustainable urban development, fuzzy AHP, fuzzy TOPSIS

## Abstract

Sustainable urban development (SUD) requires a balance between economic growth, social well-being, and environmental protection. Oftentimes, urban policy makers can hardly fulfill all SUD goals due to inadequacy of resources to support SUD programs. Therefore, the process of allocating scarce resources to achieve and balance various SUD goals becomes a critical challenge for policy makers and researchers. To solve this problem, this study adopts fuzzy Analytic Hierarchy Process (AHP) and fuzzy Technique for Order Preference by Similarity to Ideal Situation (TOPSIS) to assess and rank different indicators of SUD and evaluate different cities in an emerging economy (Vietnam). Fuzzy methods exhibit more advantages than traditional ranking methods. Fuzzy AHP is an extension of AHP, whereas fuzzy TOPSIS is an extension of TOPSIS. Fuzzy methods are used to overcome disadvantages of traditional methods and are beneficial techniques for solving complicated decision problems with a realistic solution. Using a valid sample data of ten experts in the field of SUD, empirical results show that education, healthcare, quality of life, and social democracy are the most important indicators of SUD. By contrast, social diversity, social maturity, and energy consumption are the least important indicators of SUD. For social sustainability, social democracy and quality of life are the two most important criteria, whereas social maturity and social diversity are the two least important criteria. For economic sustainability, education and healthcare are the two most important criteria, whereas infrastructure and income are the two least important criteria. For environmental sustainability, water quality and waste disposal are the two most important criteria, whereas energy consumption and ecological conservation are the two least important criteria. Furthermore, fuzzy TOPSIS results reveal the best and the worst cities in Vietnam with regard to overall SUD and its three components. This study provides evidence for researchers and policy makers to better understand the importance of different goals of SUD and efficiently allocate scarce resources to achieve and balance different SUD goals. Furthermore, researchers and policy makers should further focus on indicators such as social democracy, quality of life, education, healthcare, water quality, and waste disposal. These indicators will help obtain the goals of SUD.

## 1. Introduction

The world has witnessed several severe problems, including poverty, crimes, terrorism, climate change, pollution, and natural disasters, among others, during the last decades. These problems have encouraged people to rethink about countries’ development models. Scholars have shown interest in the relationship between economic growth and environmental protection [1]. In 1980, the International Union for the Conservation of Nature proposed the concept of “sustainable development” which is defined as “development that meets the needs of the present without compromising the ability of future generations to meet their own needs”. Since then, sustainable development has been a core focus of governments, policy makers, business managers, and researchers around the world. In September 2015, the United Nations General Assembly proposed 17 sustainable development goals that serve as guidelines for every country to achieve their sustainable objectives. Based on this foundation, researchers have extended the concept of sustainable development to every aspect of life, such as sustainability in education, business, tourism, politics, culture, and urban and rural development.

Sustainable urban development (SUD) is defined as “a process of synergetic integration, interaction, and co-evolution among the economic, social, physical and environmental subsystems making up a city which guarantees a non-decreasing level of well-being for the city population in the long term while maintaining a balance with the surrounding areas as well as contributing to reducing the harmful effects on the biosphere” [2]. SUD primarily aims to balance economic growth, social well-being, and environmental protection [3]. This process requires policy makers to take multiple components of sustainable development simultaneously into planning and implementing urban development policy. However, inadequate resources often limit urban policy makers to support SUD programs. Thus, to fulfill SUD, substantial resources are required to achieve each sustainability goal. For example, a huge amount of capital and extensive labor are needed for economic growth. Resources are also needed to create and maintain social well-being and the environment. Furthermore, different SUD goals seem to contradict one another; for instance, economic growth may cause environmental degradation and human happiness reduction at times. Therefore, the process of allocating scarce resources to achieve and balance different SUD goals has become a critical challenge for policy makers and researchers. To solve this problem, this study aims to assess and rank different indicators of SUD. The findings of this study will provide evidence for policy makers to allocate and utilize resources efficiently to achieve SUD goals. Furthermore, evaluating different cities in terms of SUD will help policy makers to identify the best city for model city building and reduce differences among cities, thereby enhancing SUD in a country.

The issue of sustainable development has become increasingly important in developing countries. Emerging countries have often focused merely on economic development [3], thus possibly ignoring or underestimating the impact of social and environmental factors on economic growth and human life [4]. However, social media has reported that overemphasizing economic development may sacrifice ecological systems and the quality of life, which may cause disasters for future generations [5]. Certain emerging economies have become the largest manufacturing bases in the world. For example, China has become the world’s largest emerging market with 54% of the population living in cities [3]. However, the country’s economic growth has caused severe pollution and environment devastation [6]. Similar to China, Vietnam has undergone rapid economic development, with an average GDP growth rate of 6.19% from 2000–2016. Vietnam has been evaluated as the second-highest growing economy in the Asia Pacific region, following China in the rank [7]. Vietnam has undergone a transition from central planning to market socialism [8]. During this period, Vietnam may experience changes in the industrial structure of the economy [9]. Meanwhile, the country has suffered from several natural disasters and pollution in recent years. These problems have encouraged the government and policy makers to focus and provide increased efforts further to drive the country into sustainable development. Therefore, to shed new light on understanding the issue of SUD, this study provides evidence for policy makers to plan and execute good decisions in developing sustainable cities in Vietnam.

In sum, this study uses fuzzy analytic hierarchy process (AHP) to assess and rank different SUD indicators. In addition, this study also adopts fuzzy Technique for Order Preference by Similarity to Ideal Situation (TOPSIS) to evaluate different cities in an emerging country. Findings of this study will help policy makers to efficiently allocate and utilize resources for the achievement of SUD goals, the development of model city, and the reduction of differences in terms of SUD among cities.

Following the Introduction section, Section 2 presents the theoretical background. Section 3 covers the study methodology, and Section 4 discusses the results and findings. Furthermore, Section 5 tackles the conclusions and limitations and proposes directions for future research.

## 2. Theoretical Background

### 2.1. Sustainable Development and Sustainable Urban Development

The United Nations [10] defines sustainable development (SD) as the development that balances the needs of present and future generations. That is, SD fulfills the needs of the present generation without compromising the ability of future generations to meet their needs. SD is a complex concept that involves the interaction among economic, social, and environmental aspects [11]. First, economic sustainability refers to “a generating income and stability for society members without the erosion of capital and resources” [12]. Sustainable economic development aims to increase jobs and revenues and create and implement strategies that balance social equity and environment alongside economic growth [13]. Economic sustainability reflects three core relationships, namely, justice among people of the same generation, justice among people of different generations, and justice between human and nature [14]. Second, social sustainability focuses on democracy, equity, and well-being [15]. Chiu [16] describes that the main purpose of social sustainability is enlightening and sustaining human welfare. Murphy [17] suggested that social sustainability reflects two specific characteristics, namely, equity and social cohesion. Davoodi et al. [18] used six dimensions, including social security, social flexibility, social hierarchy, social interaction, social architectural identity, and social participatory design, to describe sustainable social development. Finally, environmental sustainability refers to the protection of environment and improvement of resources and biodiversity to ensure that the natural resources needed for life are unimpaired and available for future generations [19]. Environmental sustainability requires great efforts to solve several environmental problems such as pollution, climate change, fossil fuel scarcity, and deforestation, among others [20].

Based on the concept of SD, several researchers have determined SUD from the three major areas of social, economic, and environmental dimensions [20]. The SD of a city maintains harmony among social, economic, and environmental development [3]. Tran [2] summarized 85 indicators of SUD and used clustering and multivariate linear regression to select a set of important indicators. Yang et al. [3] employed the linear dimensionless analysis method to investigate influencing factors of SD in 287 cities in China. Ameen and Mourshed [21] integrated 18 indicators of SUD and use the AHP method to rank these indicators. Cease et al. [4] analyzed barriers and incentives for sustainable urban development. The authors identify four key dimensions of SUD, namely, economic, policy, public awareness, and organizational. Verma and Raghubanshi [20] suggested that SUD should be assessed in eight dimensions, including: (1) environmental protection, (2) transportation, (3) quality of life, (4) green economy (e.g., clean technology and green tax policy), (5) renewable energy and waste management, (6) energy consumption, (7) preservation of cultural and natural heritage, and (8) environmental justice and equity. Moroke et al. [22] also evaluated SUD in terms of spatial, density, connectivity and mobility, culture and social capital, economic, smart growth, services, food security, governance, and livability factors. Although previous studies have determined SUD using different indicators, Ameen and Mourshed [21] concluded that previous assessments of SUD can be classified into three main categories, namely, economic, social, and environmental dimensions.

### 2.2. Assessment Methods

Researchers have used several assessment tools to evaluate SUD. Kaur and Garg [23] reviewed 2594 articles and identified the six most widely used assessment tools of SUD, namely, Building Research Establishment Environmental Assessment Method for Communities (BREEAM Communities), Comprehensive Assessment System for Built Environment Efficiency for Urban Development (CASBEE-UD), Green Building Index for Township (GBI Township), Leadership in Energy and Environmental Design for Neighborhood Development (LEED-ND), Indian Green Building Council for Green Townships (IGBC Green Township), and Green Rating for Integrated Habitat Assessment for Large Development (GRIHA-LD) (see [23] for details). Moreover, researchers have used the Multicriteria Decision Making Tool (MCDM) to select and rate indicators of SUD [22,24]. Santos et al. [11] reviewed 376 papers published from 2014–2018 and indexed in Web of Science, Scopus, and Science Direct; they concluded that AHP is one of the most widely used methods to assess SD. Given a wide range of method used in previous studies, this study adopts fuzzy AHP and fuzzy TOPSIS to assess sustainable urban development.

#### 2.2.1. Fuzzy AHP

Saaty [25] proposed the AHP method, which is the most frequently used tool for decisions based on multicriteria decision making. Given that AHP exhibits drawbacks owing to its use of exact crisp numbers (e.g., 1–9) to determine the importance of criteria, fuzzy AHP is developed as an AHP extension which overcomes the drawbacks of AHP. Experts adopt natural linguistic terms (e.g., equally important, weakly important) to express their judgments in fuzzy AHP (see Table 1).

● *Step 1*: Construct fuzzy pairwise comparison matrices.

(1) $${\tilde{A}}^{k}\;=\;\left[\begin{array}{cccc} {\tilde{d}}_{11}^{k} & {\tilde{d}}_{12}^{k} & \ldots & {\tilde{d}}_{1n}^{k}\\ {\tilde{d}}_{21}^{k} & \ldots & \ldots & {\tilde{d}}_{2n}^{k}\\ \ldots & \ldots & \ldots & \ldots \\ {\tilde{d}}_{n1}^{k} & {\tilde{d}}_{n2}^{k} & \ldots & {\tilde{d}}_{nn}^{k} \end{array}\right]\;$$

where $\tilde{d_{ij}^{k}}$ represents the *k*th decision makers’ preference of the *i*th criterion over the *j*th criterion. If several decision makers evaluate their judgment, then an averaged preference for pairwise comparison matrices for all criteria is updated as follows:

(2) $$\tilde{A}=\;\left[\begin{array}{ccc} \tilde{d_{11}} & \cdots & \tilde{d_{1n}}\\ \cdots & \ddots & \ldots \\ \tilde{d_{n1}} & \cdots & \tilde{d_{nn}} \end{array}\right]$$

where:

(3) $$\tilde{d_{ij}}\;=\;\frac{\sum_{k\;=\;1}^{k}{\tilde{d}}_{ij}^{k}}{K}$$

● *Step 2*: Use the geometrical mean technique to define the fuzzy geometrical mean and fuzzy weights of each criterion.

(4) $${\tilde{r}}_{i}={\left(\overset{n}{\underset{j\;=\;1}{\prod }}{\tilde{d}}_{ij}\right)}^{1/n},\;i\;=\;1,2,\ldots ,n$$

where ${\tilde{r}}_{i}$ is the fuzzy geometrical mean and $\tilde{d_{ij}}$ is the decision makers’ preference of the *i*th criterion over the *j*th criterion.

● *Step 3*: Determine the fuzzy weight of the criteria.

(5) $${\tilde{w}}_{i}={\tilde{r}}_{i}\times {\left({\tilde{r}}_{1}+{\tilde{r}}_{2}+\cdots +{\tilde{r}}_{n}\right)}^{-1}$$

where ${\tilde{w}}_{i}$ is the fuzzy weight of the criteria.

● *Step 4*: Calculate the average and normalized weight criteria:

(6) $$M_{i}\;=\;\frac{{\tilde{w}}_{1}+{\tilde{w}}_{2}+\cdots +{\tilde{w}}_{n}}{n}$$

(7) $$N_{i}\;=\;\frac{M_{i}}{M_{1}+M_{2}+\cdots +M_{n}}\;$$

where $M_{i}$ is the average, and $N_{i}$ is the normalized weight criteria.

#### 2.2.2. Fuzzy TOPSIS

Fuzzy TOPSIS is often used to rank different alternatives based on the shortest and farthest distances from the positive-ideal solution and the negative-ideal solution, respectively. Shaverdi et al. [27] and Chou et al. [28] note that fuzzy TOPSIS is calculated by the following steps.

● *Step 1*: Determine the weights of the evaluation criteria.

Weights of criteria from fuzzy AHP are used for fuzzy TOPSIS.

● *Step 2*: Construct the fuzzy decision matrix:

(8) $$\tilde{D}=\;\begin{array}{c} A_{1}\\ \ldots \\ A_{m} \end{array}\begin{array}{c} C_{1}\;\ldots \;C_{n}\\ \left[\begin{array}{ccc} {\tilde{x}}_{11} & \cdots & {\tilde{x}}_{1n}\\ \vdots & \ddots & \vdots \\ {\tilde{x}}_{m1} & \cdots & {\tilde{x}}_{mn} \end{array}\right] \end{array},\;i=1,2,\ldots ,m;j=1,2,\ldots ,n$$

where ${\tilde{x}}_{ij}^{k}=\left(a_{ij},b_{ij},c_{ij}\right)$, $a_{ij}=\;min_{k}\;\left\{a_{ij}^{k}\right\}\;$, $b_{ij}=\frac{1}{k}\overset{k}{\underset{k=1}{\sum }}b_{ij}^{k}$, $c_{ij}=max_{k}\;\left\{c_{ij}^{k}\right\}$

${\tilde{x}}_{ij}^{k}$ is the performance rating of the alternative $A_{i}$ with respect to criterion $C_{j}$ evaluated by the _k_^th^ expert.

● *Step 3*: Compute the normalized fuzzy decision matrix.

(9) $${\tilde{r}}_{ij}\;=\;\left(\frac{a_{ij}}{c_{j}^{*}},\frac{b_{ij}}{c_{j}^{*}},\frac{c_{ij}}{c_{j}^{*}}\right)\;and\;c_{j}^{*}\;=\;max_{i}\left\{c_{ij}\right\}\;for\;benefit\;criteria$$

(10) $${\tilde{r}}_{ij}=\;\left(\frac{a_{j}{}^{-}}{c_{ij}},\frac{a_{j}{}^{-}}{b_{ij}},\frac{a_{j}{}^{-}}{a_{ij}}\right)\;and\;a_{j}{}^{-}\;=\;min_{i}\left\{a_{ij}\right\}\;for\;cost\;criteria$$

Then, compute the weighted normalized fuzzy decision matrix

(11) $${\tilde{v}}_{ij}={\tilde{r}}_{ij}\times w_{j}\;$$

where ${\tilde{v}}_{ij}$ is the weighted normalized fuzzy decision matrix.

● *Step 4*: Determine the fuzzy positive-ideal solution (FPIS) and fuzzy negative-ideal solution (FNIS).

(12) $$A^{+}=\left({\tilde{v}}_{1}^{*},{\tilde{v}}_{2}^{*},\ldots ,{\tilde{v}}_{n}^{*}\right),\;where\;{\tilde{v}}_{j}^{*}=max_{i}\left\{v_{ij3}\right\}$$

(13) $$A^{-}=\left({\tilde{v}}_{1}^{-},{\tilde{v}}_{2}^{-},\ldots ,{\tilde{v}}_{n}^{-}\right),\;where\;{\tilde{v}}_{j}^{-}=min_{i}\left\{v_{ij1}\right\}$$

where $A^{+}$ is the fuzzy positive-ideal solution and $A^{-}$ is the fuzzy negative-ideal solution.

● *Step 5*: Calculate the distance of each alternative from FPIS and FNIS.

The distances (v) of each alternative from *A*^+^ and *A^−^* can be calculated using the area compensation method:

(14) $${\tilde{d}}_{i}^{+}=\sum_{j=1}^{n}d({\tilde{v}}_{ij},{\tilde{v}}_{j}^{*}),i=1,2,\ldots ,m;\;j=1,2,\ldots ,n$$

(15) $${\tilde{d}}_{i}^{-}=\sum_{j=1}^{n}d({\tilde{v}}_{ij},{\tilde{v}}_{j}^{-}),i=1,2,\ldots ,m;\;j=1,2,\ldots ,n$$

where ${\tilde{d}}_{i}^{+}$ and ${\tilde{d}}_{i}^{-}$ are the distances from *A*^+^ and *A^−^.*

● *Step 6*: Obtain the closeness coefficients (relative gaps–degree) and improve the alternatives to achieve the aspiration levels in each criterion:

(16) $$C{\tilde{C}}_{i}=\frac{{\tilde{d}}_{i}^{-}}{{\tilde{d}}_{i}^{+}+{\tilde{d}}_{i}^{-}}=1-\frac{{\tilde{d}}_{i}^{+}}{{\tilde{d}}_{i}^{+}+{\tilde{d}}_{i}^{-}},\;i=1,2,\ldots ,m$$

where $C{\tilde{C}}_{i}$ is the closeness coefficients.

## 3. Materials and Data

### 3.1. Assessing Criteria and Alternatives

Based on a summary of indicators proposed by Tran [2] and Santos et al. [11] and drawing from the Sustainable Development Goals of the United Nations [29], this study determines social, economic, and environmental sustainability as three components of SUD. Experts from an SUD association in Vietnam were invited to select the most important indicators of SUD from a list summarized by Tran [2] and Santos et al. [11] and from the Sustainable Development Goals of the United Nations [29]. The most frequently selected criteria are chosen based on experts’ judgment, and a set of 20 indicators are selected as criteria for assessment in this study. Given that the criteria are summarized by Tran [2] and Santos et al. [11] from more than 500 papers published from 2014–2018 in the field of SUD, these criteria are viewed as reliable indicators of SUD [2,11].

**Figure 1:**
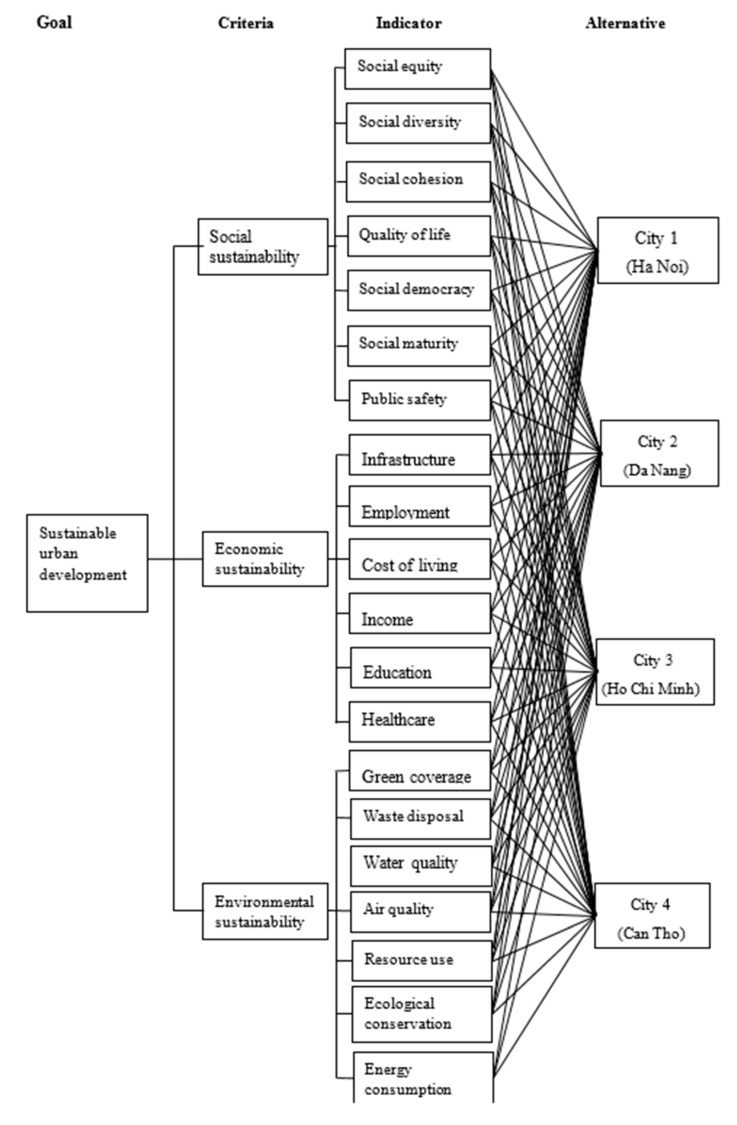
Criteria of sustainable urban development.

Social sustainability comprises seven indicators, namely, social equity, social diversity, social cohesion, quality of life, social democracy, social maturity, and public safety. Economic sustainability contains six indicators including employment, income, cost of living, infrastructure, education, and healthcare. Environmental sustainability consists of seven indicators, namely, resource use, waste disposal, water quality, air quality, green coverage, ecological conservation, and energy consumption. Figure 1 shows the criteria and indicators of SUD in this study.

A set of major cities in Vietnam is also chosen as assessment alternatives in this study. These cities are Ha Noi, Ho Chi Minh, Da Nang, and Can Tho. They are the largest and most developed cities in Vietnam (Ha Noi is the capital of Vietnam, Ho Chi Minh city is the largest city in Vietnam, Da Nang is the largest city in the central region of Vietnam, and Can Tho is the biggest city in the southern region of Vietnam). Furthermore, Vietnam implements a focused development strategy that invests all resources to certain central cities of the country. Ha Noi, Ho Chi Minh, Da Nang, and Can Tho are the targets of this strategy. Currently, these cities are also selected as the focus of SUD programs in Vietnam. These cities play as role model cities for planning and implementing SUD programs. Therefore, we select these four cities as the sample of this study. Table 2 shows some important descriptive statistics of the target cities.

### 3.2. Data collection

**Figure 2:**
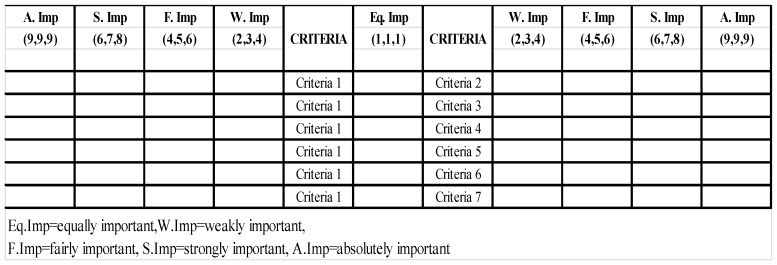
Example of pairwise comparison between criteria in questionnaire.

A list of experts who are committee members of a SUD association in Vietnam served as the sample population. A computer generator was used to generate a random table of numbers, and a simple random sampling technique was used to select respondents. A total of 16 out of 36 experts were randomly selected as a sample and were invited to complete a questionnaire. Before the formal survey, we invited the experts to select the most important criteria of SUD from a list summarized by Tran [2] and Santos et al. [11]. The only indicators that exhibit the highest frequency of selection are chosen as indicators in this study. Pairwise comparison matrices among indicators were conducted to form a questionnaire (see Figure 2). This questionnaire was designed by using forward and backward translation from English to Vietnamese and vice versa. A pilot test was conducted with five Master and PhD. students to ensure the clarity and meanings of the criteria. After revision, experts, were subsequently asked to complete the questionnaire. Six experts did not respond to our invitation, thus only ten experts completed the questionnaire, which were all considered valid. Demographic characteristics of the respondents show that six experts were males, whereas four were females. Their ages ranged from 31–50, and their average income was approximately 600 USD. All experts hold master or higher degrees. Furthermore, all of them had at least five years of experience in the field.

Tran [2], Cease et al. [4], Santos et al. [11], Ameen and Mourshed [21], and Satty [25] suggest that a small sample is appropriate for the use of fuzzy methods given that a large sample may lead to more complex and tedious computation. Satty [25] also recommends that an ideal sample should be less than twenty. Thus, a sample data of sixteen with ten valid respondents is acceptable in this study.

### 3.3. Methods

Multi-Criteria Decision Making (MCDM) is one of the most important techniques that deal with the problem involving multiple and conflicting objectives. The purpose of MCDM is to provide an overall ordering of options. MCDM also provides a systematic procedure to help decision makers select the most desirable alternative under uncertain and complex situation [22,24]. In the field of SUD, most researchers have used the traditional AHP and TOPSIS methods to assess sustainable development problems [11,23]. However, experts have used crisp numbers (e.g., 1–9) to determine the importance of criteria in traditional methods. This method has led to an unrealistic solution to a problem. Furthermore, traditional methods have exhibited a subjective nature of the modeling process, which means that this methodology cannot guarantee the decisions as definitely true. To overcome these disadvantages, fuzzy methods were used to replace the traditional methods in this study. Using fuzzy methods has allowed the use of fuzzy numbers, instead of precise numbers, which can be assigned to represent the relative importance of attributes. The use of fuzzy numbers is consistent with the real-world fuzzy environment. Fuzzy methods have been particularly suitable for solving group decision-making problems under fuzzy environments. In addition, the aim of this study is to rank indicators of SUD and select the best alternatives in a multiple and complex problem. Therefore, to ensure a more realistic solution that is suitable for the purpose of selecting the best indicators and alternatives of SUD, this study has adopted fuzzy AHP and fuzzy TOPSIS, instead of the conventional AHP and TOPSIS, to determine the issue of SUD.

## 4. Empirical Results

### 4.1. Results of Fuzzy AHP

Fuzzy AHP was calculated for three components of SUD, namely, social, economic, and environmental sustainability. Results of overall SUD were also computed. We demonstrated the calculation process for social sustainability in the following section. Results for economic, environmental, and overall SUD were shown in the next section.

● *Step 1*: Construct fuzzy pairwise comparison matrices. (see Table 3)

● *Step 2:* Use the geometrical mean technique to define the fuzzy geometrical mean and fuzzy weights of each criterion. (see Table 4)

(17) $${\tilde{r}}_{i}\;=\;{\left(\overset{n}{\underset{j\;=\;1}{\prod }}{\tilde{d}}_{ij}\right)}^{1/n}$$

Example: ${\tilde{r}}_{2}={\left(2\times 1\times 0.13\times 9\times 9\times 2\times 2\times 0.11\times 0.13\times 9\right)}^{1/10}\;=\;1.26$

● *Step 3*: Determine the fuzzy weight of the criteria. (see Table 5)

(18) $${\tilde{w}}_{i}={\;r\;˜}_{i}\times {\left({\tilde{r}}_{1}+{\tilde{r}}_{2}+\cdots +{\tilde{r}}_{n}\right)}^{-1}$$

Example: ${\tilde{R}}_{2}={\left(1.26\times 1\times 2.19\times 2.19\times 2.58\times 2.02\times 2.38\right)}^{1/7}=1.85$, ${\tilde{w}}_{2}=1.85\times 1.12=0.22$

● *Step 4:* Calculate the average and normalized weight criteria. (see Table 5)

(19) $$M_{i}\;=\;\frac{{\tilde{w}}_{1}+{\tilde{w}}_{2}+\cdots +{\tilde{w}}_{n}}{n}$$

(20) $$N_{i}\;=\;\frac{M_{i}}{M_{1}+M_{2}+\cdots +M_{n}}\;$$

Example: $M_{2}\;=\;\frac{0.22+0.27+0.33}{3}\;=\;0.27$, $N_{2}=\frac{0.27}{0.11+0.27+0.15+0.08+0.07+0.20+0.13}=0.27$

Inputs for fuzzy pairwise comparison matrices are inversely coded in this study. Results indicate that criteria ranking must be ascending (from small to large value). Table 6 shows that SS5 (social democracy) and SS4 (quality of life) are the two most important criteria of social sustainability. By contrast, SS6 (social maturity) and SS2 (social diversity) are the two least important criteria of social sustainability.

Table 7 shows the results of fuzzy AHP for economic sustainability. Results show that ECS5 (education) and ECS6 (healthcare) are the two most important criteria of economic sustainability. By contrast, ECS1 (infrastructure) and ECS4 (income) are the two least important criteria of economic sustainability.

Table 8 presents the results of fuzzy AHP for environmental sustainability. Results show that ES3 (water quality) and ES2 (waste disposal) are the two most important criteria of environmental sustainability. By contrast, ES7 (energy consumption) and ES6 (ecological conservation) are the two least important criteria of environmental sustainability.

Table 9 shows the results of fuzzy AHP for overall criteria of SUD. Results indicate that ECS5 (education), ECS6 (healthcare), SS4 (quality of life), and SS5 (social democracy) are the top four most important indicators of SUD. By contrast, SS2 (social diversity), SS6 (social maturity), and ES7 (energy consumption) are the least three important indicators of SUD.

### 4.2. Results of Fuzzy TOPSIS

Fuzzy TOPSIS was calculated for overall criteria of SUD and its three components, namely, social, economic, and environmental sustainability. We demonstrated the calculation process for economic sustainability in the following section. Results for social, environmental, and overall SUD were also shown in the next section.

● *Step 1*: Determine the weights of the evaluation criteria. (see Table 10)

Weights of all criteria were obtained from the results of fuzzy AHP in the previous section.

● *Step 2*: Construct the fuzzy decision matrix. (see Table 11)

$${\tilde{x}}_{ij}^{k}\;=\;\left(a_{ij},b_{ij},c_{ij}\right),\;a_{ij}=\;min_{k}\;\left\{a_{ij}^{k}\right\}\;,\;b_{ij}\;=\;\frac{1}{k}\sum_{k=1}^{k}b_{ij}^{k},\;c_{ij}\;=\;max_{k}\;\left\{c_{ij}^{k}\right\}\;$$

Example: ${\tilde{x}}_{11\;}^{1}=\;\left(a_{11},b_{11},c_{11}\right)=\left(2,5.2,8\right)$

where

$$a_{11}=\;min_{10}\;\left\{a_{11}^{10}\right\}\;=\;\min\left(4,6,4,4,4,4,4,6,2,4\right)=2\;b_{11}\;=\;\frac{1}{10}\overset{10}{\underset{1}{\sum }}\left(5+7+5+5+5+5+5+7+3+5\right)=5.2\;c_{11}\;=\;max_{10}\;\left\{c_{11}^{10}\right\}\;=\;\max\left(6,8,6,6,6,6,6,8,4,6\right)=8$$

● *Step 3*: Compute the normalized fuzzy decision matrix. (see Table 12)

Benefit criteria: ${\tilde{r}}_{ij}=\left(\frac{a_{ij}}{c_{j}^{*}},\frac{b_{ij}}{c_{j}^{*}},\frac{c_{ij}}{c_{j}^{*}}\right)\;and\;c_{j}^{*}=max_{i}\left\{c_{ij}\right\}$

Example: calculate ECS1: $c_{1}^{*}=max\left\{c_{i1}\right\}=\max\left(8,8,8,8\right)=8$

$${\tilde{r}}_{11}=\left(\frac{a_{11}}{c_{1}^{*}},\frac{b_{11}}{c_{1}^{*}},\frac{c_{11}}{c_{1}^{*}}\right)=\left(\frac{2}{8},\frac{5.2}{8},\frac{8}{8}\right)=\left(0.25,0.65,1\right)$$

Cost criteria: ${\tilde{r}}_{ij}=\left(\frac{a_{j}{}^{-}}{c_{ij}},\frac{a_{j}{}^{-}}{b_{ij}},\frac{a_{j}{}^{-}}{a_{ij}}\right)\;and\;a_{j}{}^{-}=min_{i}\left\{a_{ij}\right\}$

Example: calculate ECS3: $a_{3}{}^{-}=min\left\{a_{i3}\right\}=\min\left(2,2,2,2\right)=2$

$${\tilde{r}}_{13}=\left(\frac{a_{3}{}^{-}}{c_{13}},\frac{a_{3}{}^{-}}{b_{13}},\frac{a_{3}{}^{-}}{a_{13}}\right)=\left(\frac{2}{6},\frac{2}{3.6},\frac{2}{2}\right)=\left(0.33,\;0.56,\;1\right)$$

Then, compute the weighted normalized fuzzy decision matrix.

$${\tilde{v}}_{ij}={\tilde{r}}_{ij}\times w_{j}$$

Example: calculate ECS1

$${\tilde{v}}_{11}={\tilde{r}}_{11}\times w_{1}=\left(0.444,\;0.051,\;0.060\right)\times \left(0.25,\;0.65,\;1.00\right)=\left(0.011,\;0.033,\;0.060\right)$$

● *Step 4:* Determine the FPIS and FNIS. (see Table 13)

$$A^{+}\;=\;\left({\tilde{v}}_{1}^{*},{\tilde{v}}_{2}^{*},\ldots ,{\tilde{v}}_{n}^{*}\right),\;where\;{\tilde{v}}_{j}^{*}\;=\;max_{i}\left\{v_{ij3}\right\}\;A^{-}=\left({\tilde{v}}_{1}^{-},{\tilde{v}}_{2}^{-},\ldots ,{\tilde{v}}_{n}^{-}\right),\;where\;{\tilde{v}}_{j}^{-}=min_{i}\left\{v_{ij1}\right\}$$

Example: calculate ECS1

$$A^{+}{}_{1}=\left(0.02,\;0.04,\;0.06\right),\;where\;{\tilde{v}}_{1}^{*}=max\left\{0.060,\;0.060,\;0.060,\;0.060\right\}=0.060\;A^{-}{}_{1}=\left(0.01,\;0.02,\;0.06\right),\;where\;{\tilde{v}}_{1}^{*}=min\left\{0.011,\;0.022,\;0.022,\;0.011\right\}=0.011$$

● *Step 5*: Calculate the distance of each alternative from FPIS and FNIS. (see Table 13 and Table 14)

$${\tilde{d}}_{i}^{+}=\sum_{j=1}^{n}d({\tilde{v}}_{ij},{\tilde{v}}_{j}^{*}),{\tilde{d}}_{i}^{-}=\sum_{j=1}^{n}d({\tilde{v}}_{ij},{\tilde{v}}_{j}^{-})$$

Example: calculate ECS1

$${\tilde{d}}_{1}^{+}=\sqrt{\frac{1}{3}\left[{\left(0.011-0.02\right)}^{2}+{\left(0.033-0.04\right)}^{2}+{\left(0.060-0.06\right)}^{2}\right]}=0.007\;{\tilde{d}}_{1}^{-}=\sqrt{\frac{1}{3}\left[{\left(0.011-0.01\right)}^{2}+{\left(0.033-0.03\right)}^{2}+{\left(0.060-0.06\right)}^{2}\right]}=0.001$$

● *Step 6*: Obtain the closeness coefficients and improve the alternatives to achieve the aspiration levels in each criterion. (see Table 15)

$${\tilde{CC}}_{i}=\frac{{\tilde{d}}_{i}^{-}}{{\tilde{d}}_{i}^{+}+{\tilde{d}}_{i}^{-}}\;{\tilde{CC}}_{1}=\frac{0.006}{0.030+0.006}=0.176$$

Table 15 presents the ranking of four cities in terms of economic sustainability. Results show that Ho Chi Minh City ranks first, Da Nang City ranks second, Can Tho City ranks third, and Ha Noi City ranks fourth in economic sustainability.

Similarly, Table 16 shows the ranking of four cities in terms of social sustainability. Results show that Da Nang City ranks first, Ho Chi Minh City ranks second, Ha Noi City ranks third, and Can Tho City ranks fourth in social sustainability.

Table 17 shows the ranking of four cities in terms of environmental sustainability. Results show that Da Nang City ranks first, Ho Chi Minh City ranks second, Can Tho City ranks third, and Ha Noi City ranks fourth in environmental sustainability.

Table 18 shows the ranking of four cities in terms of overall SUD. Results show that Da Nang City ranks first, Ho Chi Minh City ranks second, Can Tho City ranks third, and Ha Noi City ranks fourth in overall SUD.

## 5. Discussion and Conclusions

This study uses fuzzy AHP to evaluate different SUD criteria. Empirical results show that education, healthcare, quality of life, and social democracy are the most important indicators of SUD. By contrast, social diversity, social maturity, and energy consumption are the least important indicators of SUD. Furthermore, social democracy and quality of life are the two most important criteria, whereas social maturity and social diversity are the two least important criteria of social sustainability. In addition, education and healthcare are the two most important criteria, whereas infrastructure and income are the two least important criteria of economic sustainability. Finally, water quality and waste disposal are the two most important criteria, whereas energy consumption and ecological conservation are the two least important criteria of environmental sustainability. Furthermore, this study also uses fuzzy TOPSIS to assess different cities in Vietnam based on SUD criteria. Results show that Da Nang City ranks the best, whereas Ha Noi City ranks the worst in terms of overall criteria of SUD. In terms of social sustainability, Da Nang City ranks the best, whereas Can Tho City ranks the worst. In terms of economic sustainability, Ho Chi Minh City ranks the best, whereas Ha Noi City ranks the worst. In terms of environmental sustainability, Da Nang City ranks the best, whereas Ha Noi City ranks the worst. The findings of this study provide important implications for academic researchers and practitioners.

### 5.1. Theoretical and Practical Implications

First, researchers have discussed several models of development for urban and rural areas in different countries [2]. Solely focusing on economic development may lead to ignorance or may exhibit negative impact to society and the environment [3]. Current disasters around the world call our attention to an SD model. Thus, SUD has become a core focus for policy makers and researchers in recent years [20]. SUD requires a balance among social, economic, and environmental development [1]. However, fulfilling all different SUD goals is a challenging task due to frequent scarcity of resources which cannot sufficiently support all SUD activities. Therefore, this study provides evidence for researchers and policy makers to further understand the importance of different SUD goals and efficiently allocate scarce resources to achieve and balance these goals. To our knowledge, this study is the first research that determines the important weights of SUD indicators. Our findings shed new light on policy makers and researchers to support their planning and implementation of SUD programs.

Second, social sustainability is an important component of SUD [16]. Researchers and policy makers often emphasize the importance of social security and public safety [15]. They often endeavor to build and maintain an orderly and safe society. However, our findings show that social democracy and quality of life are the two most important indicators of social sustainability. Therefore, researchers and policy makers should invest additional resources and efforts to improve social democracy and the citizens’ quality of life. A development model of social sustainability requires policy makers to focus on democracy and quality of life further. Such focus is important because citizens of any society may demand for better quality of life than what they currently experience and may call for a democratic society [20].

Third, economic development is critically important for any country. This finding emphasizes the improvement of economic growth or GDP growth [4,12]. Certain developing countries may overemphasize economic growth while sacrificing human well-being and environmental welfare [6]. Sole focus on economic growth may lead to ignorance or may yield insufficient investment on other aspects of an economy (e.g., education, health service, and employment). However, our findings show that education and healthcare are the two most important goals of economic sustainability. This finding implies that researchers and policy makers should pay more attention to improve the education and healthcare services for citizens. Economic growth may be an important indicator, but education and healthcare are even more important goals for the SD of an economy. This finding implies that researchers and policy makers should take education and healthcare into their policies to build and maintain a sustainable economy.

The importance of environmental protection is often underestimated in planning and implementing SUD [20]. However, climate change and natural disasters have led to an increasing interest on environmental protection in the last decades. This study finds that water quality and waste disposal are the two most important criteria of environmental sustainability, which reflects that these criteria are the highest concerns of the citizens in any country. Social media has reported that the lack of water and the worsening of water pollution are serious problems worldwide [20]. Consumption society has also created billions of tons of waste that is released to environment. This waste concern has caused serious pollution and has posed negative impacts to human health and the natural environment [4]. Therefore, to protect the environment and build a better world, researchers and policy makers should invest additional amount of money and exert further efforts to improve water quality and reduce and dispose waste properly.

The results of fuzzy TOPSIS reveal that Da Nang is the best city in Vietnam in terms of SUD. Our findings imply that researchers and policy makers in Vietnam should consider the SD model of Da Nang City as it ranks first in terms of overall indicators of SUD. The results also indicate that Da Nang City ranks first in terms of social and environmental sustainability. This finding implies that the development policy of Da Nang City is effective and can play as a role model for other cities in Vietnam. In addition, we find that Ho Chi Minh City ranks first in terms of economic sustainability. This finding indicates that other cities in Vietnam should learn from the case of Ho Chi Minh City in planning and implementing SUD. To build a sustainable city, researchers and policy makers should understand the importance of different SUD indicators and consider the differences among the cities in a country. Identifying the most indicators of SUD and the country’s best city will help researchers and policy makers to improve their decision-making process and build an ideal city.

Finally, Vietnam is an emerging country and is considered one of the numerous high-potential Asia-Pacific markets. Vietnam has recently gone through rapid economic development with an average GDP growth rate of 6.19% from 2000–2016. The country has been evaluated as the second highest-growing economy in the Asia-Pacific region, following China in the ranking. The findings of this study may provide important implications for other cities in emerging economies worldwide given that many emerging and third world countries may undergo a similar development process to Vietnam. Moving from economic development to an SD model requires policy makers and government agencies to take the case of other cities into their decision making. Furthermore, they may refer to this study’s findings.

### 5.2. Limitations and Future Research

We acknowledge that this study has several limitations that should be improved in future research. Sample data has been collected from ten experts in the field of SUD, which is a reasonable sample size for the use of fuzzy AHP and fuzzy TOPSIS. However, future research should collect data from a larger number of experts from different social science fields (e.g., urban development experts, economic experts, social development experts, and environmental science experts). Moreover, this study solely considered Vietnam in the context of an emerging economy. The findings may limit the generalization of this study. Thus, future research should consider other emerging economies, such as China, Russia, India, and other Southeast Asian countries. In addition, this study used fuzzy AHP and fuzzy TOPOSIS to evaluate and rank different indicators and different cities of SUD. Antecedents and consequences of SUD were not determined. Therefore, future research should use other methods (e.g., regression analysis) to examine the cause and effect of different SUD indicators.

## Figures and Tables

**Table 1:** Linguistic terms of fuzzy AHP.

Saaty Scale	Definition	Fuzzy Triangle Scale
1	Equally important	(1, 1, 1)
3	Weakly important	(2, 3, 4)
5	Fairly important	(4, 5, 6)
7	Strongly important	(6, 7, 8)
9	Absolutely important	(9, 9, 9)
2	Intermittent values between two adjacent scales	(1, 2, 3)
4		(3, 4, 5)
6		(5, 6, 7)
8		(7, 8, 9)

Chang [26] introduces the following steps of fuzzy AHP.

**Table 2:** Descriptive statistics of major cities in Vietnam (2018).

Characteristics/Cities	Ha Noi	Ho Chi Minh	Da Nang	Can Tho
Area	3328.9 km^2^	2061.2 km^2^	1285.4 km^2^	1408.9 km^2^
Population	7,781,631	8,636,899	1,230,847	1,569,301
Population density	2300/km^2^	4097/km^2^	957/km^2^	1100/km^2^
Population growth	1.5%	1.9%	1.8%	0.8%
Literacy ratio	98.7%	98.5%	98.2%	95.4%
Labor force	3.83 million	4.42 million	0.61 million	0.73 million
GDP (nominal)	40.1 billion USD	60.83 billion USD	3.733 billion USD	3.6 billion USD
GDP per capital	5080 USD	7089 USD	3035 USD	2980 USD
GDP growth	7.2%	8%	7.86%	7.60%
Competitiveness index	65.4 (rank no. 9)	65.3 (rank no. 10)	65.7 (rank no. 5)	65.0 (rank no. 11)
FDI (USD)	33.2 billion	45.1 billion	5.2 billion	686.4 million

Source: Wikipedia.org (July 2019) and General Statistics Office of Vietnam (July 2019).

**Table 3:** Pairwise comparison matrix for Respondent 1.

Criteria	SS1			SS2			SS3			SS4			SS5			SS6			SS7		
SS1	1.00	1.00	1.00	0.25	0.33	0.50	0.17	0.20	0.25	1	1	1	1	1	1	0.17	0.20	0.25	1	1	1
SS2	2	3	4	1.00	1.00	1.00	4	5	6	4	5	6	6	7	8	4	5	6	4	5	6
SS3	4.00	5.00	6.00	0.17	0.2	0.25	1.00	1.00	1.00	4	5	6	4	5	6	4	5	6	4	5	6
SS4	1	1	1	0.17	0.2	0.25	0.17	0.2	0.25	1.00	1.00	1.00	1	1.00	1	1	1	1	1	1	1
SS5	1	1	1	0.13	0.14	0.17	0.17	0.2	0.25	1	1	1	1.00	1.00	1.00	0.25	0.33	0.25	0.25	0.33	0.25
SS6	4	5	6	0.17	0.2	0.25	0.17	0.2	0.25	1	1	1	4	3	4	1.00	1.00	1.00	1	1	1
SS7	1	1	1	0.17	0.2	0.25	0.17	0.2	0.25	1	1	1	4	3	4	1	1	1	1.00	1.00	1.00

**Table 4:** Pairwise comparison matrices for all respondents.

Criteria	SS1			SS2			SS3			SS4			SS5			SS6			SS7		
SS1	1.00	1.00	1.00	0.61	0.68	0.79	0.58	0.65	0.75	0.81	0.87	0.94	1.55	1.55	1.55	0.68	0.75	0.84	0.74	0.76	0.77
SS2	1.26	1.46	1.64	1.00	1.00	1.00	2.19	2.54	2.90	2.19	2.54	2.90	2.58	2.85	3.14	2.02	2.41	2.83	2.38	2.67	2.96
SS3	1.34	1.53	1.71	0.35	0.39	0.46	1.00	1.00	1.00	1.64	1.72	1.78	1.78	1.93	2.11	0.76	0.84	0.95	1.23	1.39	1.56
SS4	1.06	1.15	1.23	0.35	0.39	0.46	0.56	0.58	0.61	1.00	1.00	1.00	0.59	0.63	0.68	0.25	0.27	0.31	0.59	0.63	0.68
SS5	0.64	0.64	0.64	0.32	0.35	0.39	0.47	0.52	0.56	1.47	1.59	1.69	1.00	1.00	1.00	0.26	0.28	0.29	0.26	0.28	0.29
SS6	1.20	1.33	1.47	0.35	0.41	0.49	1.05	1.18	1.32	3.27	3.65	3.98	3.41	3.52	3.80	1.00	1.00	1.00	1.91	2.03	2.13
SS7	1.30	1.32	1.35	0.34	0.37	0.42	0.64	0.72	0.81	1.47	1.59	1.69	3.41	3.52	3.80	0.47	0.49	0.52	1.00	1.00	1.00

**Table 5:** Results of the fuzzy weight of all criteria for social sustainability.

Criteria	SS1	SS2	SS3	SS4	SS5	SS6	SS7	Total	R(−)		Criteria	SS1	SS2	SS3	SS4	SS5	SS6	SS7
**Ri**	0.81	1.85	1.03	0.56	0.52	1.38	0.94	7.10	0.14	0.12	Wi	0.10	0.22	0.12	0.07	0.06	0.16	0.11
	0.86	2.09	1.13	0.60	0.55	1.50	1.00	7.72	0.13	0.13		0.11	0.27	0.15	0.08	0.07	0.19	0.13
	0.92	2.32	1.23	0.65	0.57	1.63	1.06	8.39	0.12	0.14		0.13	0.33	0.17	0.09	0.08	0.23	0.15

**Table 6:** The average, normalized weight, and rank of criteria for social sustainability.

Criteria	SS1	SS2	SS3	SS4	SS5	SS6	SS7	Total
Mi	0.11	0.27	0.15	0.08	0.07	0.20	0.13	1.01
Ni	0.111	0.270	0.146	0.078	0.070	0.194	0.129	
Rank	3	7	5	2	1	6	4	

**Table 7:** The average, normalized weight, and rank of criteria for economic sustainability.

Criteria	ECS1	ECS2	ECS3	ECS4	ECS5	ECS6	Total
Mi	0.20	0.15	0.18	0.19	0.10	0.14	0.97
Ni	0.207	0.155	0.190	0.191	0.108	0.149	
Rank	6	3	4	5	1	2	

**Table 8:** The average, normalized weight, and rank of criteria for environmental sustainability.

Criteria	ES1	ES2	ES3	ES4	ES5	ES6	ES7	Total
Mi	0.14	0.12	0.11	0.12	0.15	0.16	0.21	1.01
Ni	0.137	0.120	0.106	0.121	0.144	0.161	0.211	
Rank	4	2	1	3	5	6	7	

**Table 9:** The average, normalized weight, and rank of criteria for SUD.

Criteria	SS1	SS2	SS3	SS4	SS5	SS6	SS7	ECS1	ECS2	ECS3	ECS4	ECS5	ECS6	ES1	ES2	ES3	ES4	ES5	ES6	ES7	Total
Mi	0.05	0.11	0.06	0.03	0.03	0.09	0.05	0.05	0.05	0.05	0.05	0.02	0.03	0.05	0.04	0.04	0.04	0.05	0.05	0.07	1.01
Ni	0.049	0.113	0.063	0.027	0.027	0.085	0.051	0.051	0.045	0.054	0.047	0.020	0.026	0.048	0.044	0.039	0.044	0.054	0.047	0.065	
Rank	9	15	12	3	3	14	10	10	6	11	7	1	2	8	5	4	5	11	7	13	

**Table 10:** Weights of criteria of economic sustainability.

Criteria	ECS1	ECS2	ECS3	ECS4	ECS5	ECS6
**Wi**	0.04	0.04	0.04	0.04	0.02	0.02
	0.05	0.04	0.05	0.05	0.02	0.03
	0.06	0.05	0.07	0.06	0.02	0.03

Note: ECS1 = infrastructure; ECS2 = employment; ECS3 = cost of living; ECS4 = income; ECS5 = education; ECS6 = healthcare.

**Table 11:** Decision matrix of economic sustainability.

Cities/Criteria	ECS1			ECS2			ECS3			ECS4			ECS5			ECS6		
Ha Noi City	2	5.2	8	2	4.8	8	2	3.6	6	1	3.8	8	2	4.8	8	2	3.8	6
Dang Nang City	4	6	8	2	5.6	8	2	5.2	8	1	6.6	9	2	5.6	8	2	5.2	8
Ho Chi Minh City	4	6.2	8	2	6.2	9	2	4.6	8	2	5	8	4	6.4	8	2	5.2	8
Can Tho City	2	5	8	2	5	8	2	5	8	2	5	8	2	5.2	8	2	4.6	6

Note: ECS1 = infrastructure; ECS2 = employment; ECS3 = cost of living; ECS4 = income; ECS5 = education; and ECS6 = healthcare.

**Table 12:** Weights and normalized fuzzy decision matrix.

Cities/Criteria	ECS1			ECS2			ECS3			ECS4			ECS5			ECS6		
Wi	0.044	0.051	0.060	0.038	0.045	0.054	0.044	0.054	0.066	0.039	0.047	0.057	0.017	0.020	0.023	0.022	0.026	0.032
Ha Noi City	0.25	0.65	1.00	0.22	0.53	0.89	0.33	0.56	1.00	0.11	0.42	0.89	0.25	0.60	1.00	0.25	0.475	0.75
Dang Nang City	0.50	0.75	1.00	0.22	0.62	0.89	0.25	0.39	1.00	0.11	0.73	1.00	0.25	0.70	1.00	0.25	0.65	1.00
Ho Chi Minh City	0.50	0.78	1.00	0.22	0.69	1.00	0.25	0.44	1.00	0.22	0.56	0.89	0.50	0.80	1.00	0.25	0.65	1.00
Can Tho City	0.25	0.63	1.00	0.22	0.56	0.89	0.25	0.40	1.00	0.22	0.56	0.89	0.25	0.65	1.00	0.25	0.575	0.75

Note: ECS1 = infrastructure; ECS2 = employment; ECS3 = cost of living; ECS4 = income; ECS5 = education; and ECS6 = healthcare.

**Table 13:** Normalized fuzzy decision matrix, fuzzy positive-ideal, and fuzzy negative-ideal solution.

Cities/Criteria	ECS1			ECS2			ECS3			ECS4			ECS5			ECS6		
Ha Noi City	0.011	0.033	0.060	0.008	0.024	0.048	0.015	0.030	0.066	0.004	0.020	0.051	0.004	0.012	0.023	0.006	0.012	0.024
Dang Nang City	0.022	0.038	0.060	0.008	0.028	0.048	0.011	0.021	0.066	0.004	0.035	0.057	0.004	0.014	0.023	0.006	0.017	0.032
Ho Chi Minh City	0.022	0.040	0.060	0.008	0.031	0.054	0.011	0.024	0.066	0.009	0.026	0.051	0.008	0.016	0.023	0.006	0.017	0.032
Can Tho City	0.011	0.032	0.060	0.008	0.025	0.048	0.011	0.022	0.066	0.009	0.026	0.051	0.004	0.013	0.023	0.006	0.015	0.024
A+	0.02	0.04	0.06	0.01	0.03	0.05	0.01	0.03	0.07	0.00	0.03	0.06	0.01	0.02	0.02	0.01	0.02	0.03
A-	0.01	0.03	0.06	0.01	0.02	0.05	0.01	0.02	0.07	0.00	0.02	0.05	0.00	0.01	0.02	0.01	0.01	0.02

Note: ECS1 = infrastructure; ECS2 = employment; ECS3 = cost of living; ECS4 = income; ECS5 = education; and ECS6 = healthcare.

**Table 14:** Distance of each alternative from FPIS and FNIS.

**Cities/Criteria**	**ECS1**	**ECS2**	**ECS3**	**ECS4**	**ECS5**	**ECS6**	${\tilde{d}}_{i}^{+}$
Ha Noi City	0.007	0.005	0.000	0.009	0.003	0.005	0.030
Dang Nang City	0.001	0.004	0.006	0.000	0.003	0.000	0.013
Ho Chi Minh City	0.000	0.000	0.004	0.007	0.000	0.000	0.011
Can Tho City	0.008	0.005	0.005	0.007	0.003	0.005	0.032
**Cities/Criteria**	**ECS1**	**ECS2**	**ECS3**	**ECS4**	**ECS5**	**ECS6**	${\tilde{d}}_{i}^{-}$
Ha Noi City	0.001	0.000	0.006	0.000	0.000	0.000	0.006
Dang Nang City	0.007	0.002	0.000	0.009	0.001	0.005	0.025
Ho Chi Minh City	0.008	0.005	0.002	0.004	0.003	0.005	0.028
Can Tho City	0.000	0.001	0.000	0.004	0.001	0.002	0.008

Note: ECS1 = infrastructure; ECS2 = employment; ECS3 = cost of living; ECS4 = income; ECS5 = education; and ECS6 = healthcare.

**Table 15:** Closeness coefficients and rank of alternatives for economic sustainability.

Cities	${\tilde{d}}_{i}^{+}$	${\tilde{d}}_{i}^{-}$	${\tilde{CC}}_{i}$	Rank
Ha Noi City	0.030	0.006	0.176	4
Dang Nang City	0.013	0.025	0.659	2
Ho Chi Minh City	0.011	0.028	0.717	1
Can Tho City	0.032	0.008	0.190	3

**Table 16:** Closeness coefficients and rank of alternatives for social sustainability.

Cities	${\tilde{d}}_{i}^{+}$	${\tilde{d}}_{i}^{-}$	${\tilde{CC}}_{i}$	Rank
Ha Noi City	0.108	0.024	0.183	3
Dang Nang City	0.068	0.069	0.505	1
Ho Chi Minh City	0.073	0.064	0.467	2
Can Tho City	0.094	0.016	0.147	4

**Table 17:** Closeness coefficients and rank of alternatives for environmental sustainability.

Cities	${\tilde{d}}_{i}^{+}$	${\tilde{d}}_{i}^{-}$	${\tilde{CC}}_{i}$	Rank
Ha Noi City	0.121	0.007	0.053	4
Dang Nang City	0.003	0.125	0.979	1
Ho Chi Minh City	0.047	0.103	0.687	2
Can Tho City	0.050	0.098	0.664	3

**Table 18:** Closeness coefficients and rank of alternatives for environmental sustainability.

Cities	${\tilde{d}}_{i}^{+}$	${\tilde{d}}_{i}^{-}$	${\tilde{CC}}_{i}$	Rank
Ha Noi City	0.212	0.034	0.139	4
Dang Nang City	0.107	0.157	0.595	1
Ho Chi Minh City	0.134	0.143	0.517	2
Can Tho City	0.163	0.073	0.309	3
